# Anti-VCAM-1 and Anti-IL4Rα Aptamer-Conjugated Super Paramagnetic Iron Oxide Nanoparticles for Enhanced Breast Cancer Diagnosis and Therapy

**DOI:** 10.3390/molecules25153437

**Published:** 2020-07-29

**Authors:** Raja Chinnappan, Achraf Al Faraj, Anas M. Abdel Rahman, Khalid M. Abu-Salah, Fouzi Mouffouk, Mohammed Zourob

**Affiliations:** 1 Department of Chemistry, Alfaisal University, Al Zahrawi Street, Al Maather, Al Takhassusi Rd, Riyadh 11533, Saudi Arabia; rchinnappan@alfaisal.edu; 2 Department of Radiologic Sciences, Faculty of Health Sciences, American University of Science and Technology, Ashrafieh, Alfred Naccash Avenue, Beirut 1100, Lebanon; 3 Department of Genentics, King Faisal Specialist Hospital and Research Center, Zahrawi Street, Al Maather, Riyadh 12713, Saudi Arabia; aabdelrahman46@kfshrc.edu.sa; 4 Department of Biochemistry and Molecular Medicine, College of Medicine, Al Faisal University, Riyadh 11533, Saudi Arabia; 5 Department of Chemistry, Memorial University of Newfoundland, St. John’s, NL A1B 3X7, Canada; 6 Department of Nanomedicine, King Abdullah International Medical Research Center/King Saud bin Abdulaziz University for Health Sciences, King Abdulaziz Medical City, Riyadh 11481, Saudi Arabia; abu-salahkh@NGHA.MED.SA; 7 Department of Chemistry, Kuwait University, P.O. Box 5969, Safat 13060, Kuwait; fmouffouk@ku.edu.kw

**Keywords:** VCAM1 aptamer, IL4Rα aptamer, luciferase assay, tumor imaging, MRI, BLI and SPION

## Abstract

The surface protein overexpressed on cancer cells can be used as biomarkers for early detection of specific diseases. Anti-VCAM-1 and anti-IL4Rα DNA aptamers specific to VCAM-1 and IL4Rα receptors that are overexpressed in 4T1 tumor-bearing mice could be used as potential biomarker for both diagnostic and therapeutic applications in cancer biology. Cell Viability and luciferase assay of 4T1-Luc2 cancer cells in the presence of anti-VCAM-1 ssDNA or anti-IL4Rα RNA aptamers was assessed by monitoring the changes in the absorbance and the fluorescence of Alamar blue dye. The aptamer-conjugated SPIO magnetic beads, used for the selective targeting to tumor sites, were monitored using noninvasive MRI and Bioluminescence imaging (BLI). Cell viability and luciferase assays showed that both anti-VCAM-1 and anti-IL4Rα aptamers favor the depletion of cancer cells and limit tumor progression. Microscopic analyses confirmed that the target specific aptamers significantly trigger tumor cell apoptosis and limit cancer cell growth in vitro. The intravenous injection of SPIO nanoparticle-conjugated aptamers were further confirmed using noninvasive MRI and Bioluminescence imaging. Anti-VCAM1 and anti-IL4Rα aptamers, specific to VCAM-1 and IL4Rα receptors overexpressed in 4T1-Luc2 tumor-bearing mice, were used as diagnostic and therapeutic tools.

## 1. Introduction

Selective detection of tumor cells in their early stage is critically important for enhanced diagnosis and therapy of cancer. Targeting of specific biomarkers expressed on the tumor cells is considered a promising approach for enhanced cancer management. Biomarkers include patterns of gene expression or levels of a particular protein that are expressed as an indicator of pathological processes, or as a response to a therapeutic intervention [1,2]. However, the targeting and internalizing of anti-cancerous drugs to the specific tumor sites is challenging. Small molecule chemotherapeutics, such as Doxorubicin [3] and Paclitaxel [4], have no target specific distribution. The inefficient delivery of drugs to the specific tumor sites leads to low therapeutic outcomes and negative side effects in cancer patients. Nanomaterials were introduced to be among the best drug carriers allowing even distribution at the tumor site [5]. Conjugating anti-cancerous drugs with nanomaterials provides improved tumor site targeting and the penetration of the nanocarriers via the enhanced permeability and retention (EPR) effect [6]. Though nanomaterials are one order of magnitude larger than the conventional chemotherapeutic molecules, their functional modifications significantly enhance distribution within the tumor [7].

Molecular recognition of a specific biomarker using nucleic acid aptamer is considered as a potential tool to develop a biosensor for both diagnostic and therapeutic applications [8,9]. Aptamers are single-stranded DNA or RNA with a length of 40 to 100 nucleotides (5–25 kDa). They can selectively bind to their target molecules with high affinity. The secondary or tertiary structures of the aptamers are unique for each target and can form more stable complexes with the target molecule. Aptamers were used for in vivo screening and the identification of novel riboswitches [10]. Naturally, the riboswitches were found in the 5′untranslated region of mRNA, which can regulate their own expression upon binding with metabolite and other molecules [11]. In vitro screening of aptamers for various analytes, including metal ions [12], proteins [13], bacteria [14,15], viruses [16], toxins [17], and small molecules [18,19,20] have been reported. As the aptamers recognize the protein markers overexpressed on the tumor cells surface with high affinity and specificity, they were used as a molecular probe for early diagnosis and therapy, and their application can be extended to targeted drug delivery [21]. Overexpressed Vascular Endothelial Growth Factor Receptor 2 (VEGFR2) in angiogenic vessels has been detected using Superparamagnetic Iron Oxide (SPIO) nanoparticles by MRI [22]. The aptamers selected from cell-SELEX bind to the receptor proteins associated with diseases and thus inhibit their biological activity. As the cell surface proteins undergo recycling processes, ligand-induced internalization would pave the way for the development of the aptamer-based drug therapeutic industry.

For example, Vascular Cell Adhesive Molecule-1 (VCAM-1) is seemingly expressed in the lung metastatic breast cancer cells and shows that it transmits pro-survival signals when macrophages are involved. It is reported that VCAM-1 supports metastatic cells for survival and outgrowth in the leukocyte-rich lung parenchyma microenvironment. The interaction between the leukocyte α 4-integrins and VCAM-1 in breast cancer cells renders certain susceptible to pro-apoptotic signals [23]. The suppression of VCAM-1 on breast cancer cells prevents the formation of metastatic colonies in the lungs [23]. Therefore, the depletion of VCAM-1 expression is potentially important for the prevention of cancer growth and is considered as an important therapeutic target in breast cancer. Anti-VCAM-1 aptamer would be a promising candidate as a therapeutic agent to inhibit or suppress VCAM-1 expression on cancer cell surfaces. We have selected the anti-VCAM-1 aptamer, which has been investigated for the early detection of cerebrovascular inflammation by in vivo imaging technique [24].

Myeloid Derived Suppressor Cells (MDSC) and Tumor Associated Macrophages (TAM) are known to facilitate tumor growth and the propagation of growth metastasis and angiogenesis, and to suppress the anti-tumor immune response. Though there are many signaling pathways involved in MDSC suppression, IL4Rα-STAT6 pathway seems to play an important role. It is responsible for mediating the TGF-β production, arginase activity and many other processes [25]. Many drugs have been reported for the depletion of MDSC activity; however, they fail to deplete the specific target, which leads to irrelevant side effects. Therefore, it is necessary to increase the selective targeting of MDSC to improve the anti-tumor immune response and minimize the side effects. It is known that IL4Rα is responsible for the upregulation of a number of murine tumors; therefore, blocking IL4Rα signal pathways will suppress the function of MDSC.

In this article, we investigated anti-VCAM-1 ssDNA aptamer [24] and anti-IL4Rα RNA aptamer [25], conjugated to SPIO nanoparticles, for the diagnosis and treatment of breast cancer cells. This theranostic approach provides both enhanced therapeutic and diagnostic capabilities that allow for the early-stage detection of the tumors. The active targeting of the developed nanocomplexes to VCAM-1 and IL4Rα receptors were used as an effective therapeutic and diagnostic tool for murine breast cancer using noninvasive bioluminescence and Magnetic Resonance (MR) imaging. Both in vitro cell viability and bioluminescence assays, and in vivo BLI and MRI investigations confirmed that these target specific aptamers effectively blocked the VCAM-1 and IL4Rα signals and suppressed the tumor progression in 4T1-bearing mice.

## 2. Results

### 2.1. Conjugation of Aptamers with Magnetic Beads for In Vivo Studies

5′Amine functionalized anti-VCAM-1 ssDNA aptamers and anti-IL4Rα RNA aptamers were coupled with carboxylic acid functionalized PEGylated SPIO magnetic nanoparticles by EDC/NHC chemistry. The conjugated SPIONs were used for the in vivo studies, MRI and BLI. The conjugation of magnetic beads with the aptamers was confirmed by a shift in the IR vibration frequency of the carbonyl group from 1725 cm^−1^ to 1650 cm^−1^. After conjugation, the carboxylic acids were converted to amide and the IR absorption at 1725 cm^−1^ and 1650 cm^−1^ corresponded to the carbonyl group in the carboxylic acid and amide, respectively [26] (Figure S1).

### 2.2. Effect of Aptamers on Cell Growth

Biocompatible anti-VCAM-1 ssDNA full length (11R6), truncated (A11R6), or anti-IL4Rα RNA aptamers (Cl42RNA) were incubated with 4T1-Luc2 cells and the cell viability was monitored by assessing the variation in the absorption and the fluorescence intensity of alamar blue as a probe. The non-toxic alamar blue was added along with the growth media, and the real time change in the fluorescence and the absorbance of the dye indicate the metabolic activity of the cells. Alamar blue was reduced in the presence of metabolites released from the cells. The oxidized form of dye absorbs at 600 nm and shows no fluorescence, whereas the reduced form absorbs at 570 nm and demonstrates a characteristic emission at 590 nm. The shift in the absorption wavelength and the increase in fluorescence intensity at 590 nm reflect the ongoing cellular metabolic activities.

**Figure 1:**
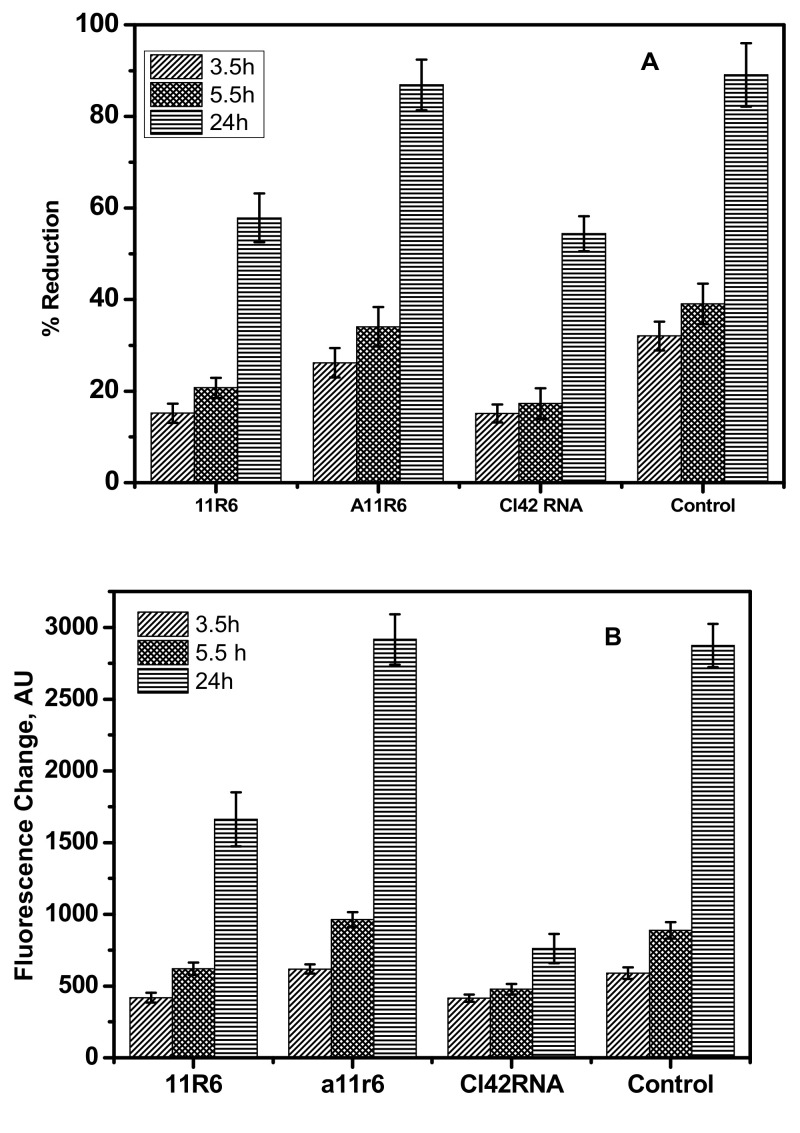
(**A**) Changes in the percentage reduction (from the absorption) in reduced Alamar blue at 570 nm in the presence of 4T1-Luc2 untreated cells or incubated with anti-VCAM-1 ssDNA full length (11R6), truncated aptamer (A11R6), or anti-IL4Rα RNA aptamers (Cl42RNA) for 3.5, 5.5 and 24 h. (**B**) Change in the fluorescence intensities of reduced alamar blue at 590 nm in the presence of 4T1-Luc2 cells untreated or incubated with anti-VCAM-1 ssDNA full length (11R6), truncated aptamer (A11R6), or anti-IL4Rα RNA aptamers (Cl42RNA) for 3.5, 5.5 and 24 h.

Cells treated with anti-VCAM-1 ssDNA or anti-IL4Rα RNA aptamers showed significant increase in the absorption at 570 nm (Figure 1A) compared to untreated cells. This is presumably due to the lower sensitivity of the UV–Visible absorption spectroscopy compared to fluorescence spectroscopy. In order to further confirm this hypothesis, more sensitive fluorescence measurements were carried out to assess the effects of aptamers on the cell growth factor. The fluorescence intensity of the reduced form of dye (Em = 590 nm) was found to increase relatively significantly, with the incubation time in the untreated cells or with truncated VCAM-1 aptamer (A11R6) (Figure 1B). However, no significant increase in the fluorescence intensity of Cl42RNA aptamers incubated with cancer cells was observed. The considerable increase in the fluorescence intensity in the presence of 11R6 compared to Cl42RNA might be due to the lower affinity of 11R6 with VCAM-1.

### 2.3. In Vitro Bioluminescence Assay

**Figure 2:**
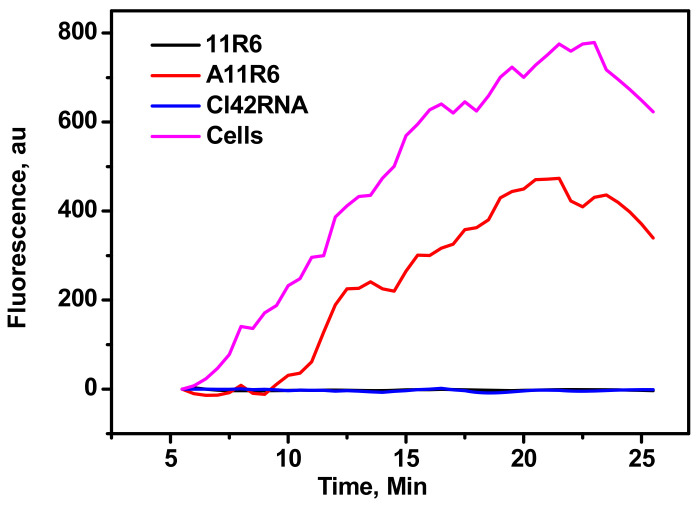
Bioluminescence assay: (Cl42RNA) overnight and the expressed luciferase enzyme was reacted with L-luciferin. Then, 4T1-Luc2 cells incubated with anti-VCAM-1 ssDNA full length (11R6), truncated aptamer (A11R6), or anti-IL4Rα RNA aptamers. Changes in the bioluminescence intensities from the treated cells are plotted with time.

As 4T1-Luc2 tumor cells carry the luciferase expressing genes, they can express the luciferase enzyme and undergo enzymatic reaction with D-luciferin, resulting in the emission of photons. Then, 4T1-Luc2 tumor cells were incubated in the presence of aptamer over night to express the luciferase. After the overnight incubation of aptamers with the cells, D-luciferin was added in the well containing 4T1-Luc2 cells with the various aptamers and the bioluminescent intensity were measured for 20 min. A moderate emission from cells incubated with A11R6 truncated VCAM-1 aptamer was observed, indicating that their influence on cell depletion was not significant. In contrast, cells incubated with the 11R6 and Cl42RNA aptamers revealed the complete absence of luminescence confirming the effective suppression of cell growth (Figure 2).

### 2.4. Effect of Anti-VCAM-1 and Anti-IL4Rα Aptamers on 4T1 Cells Apoptosis

**Figure 3:**
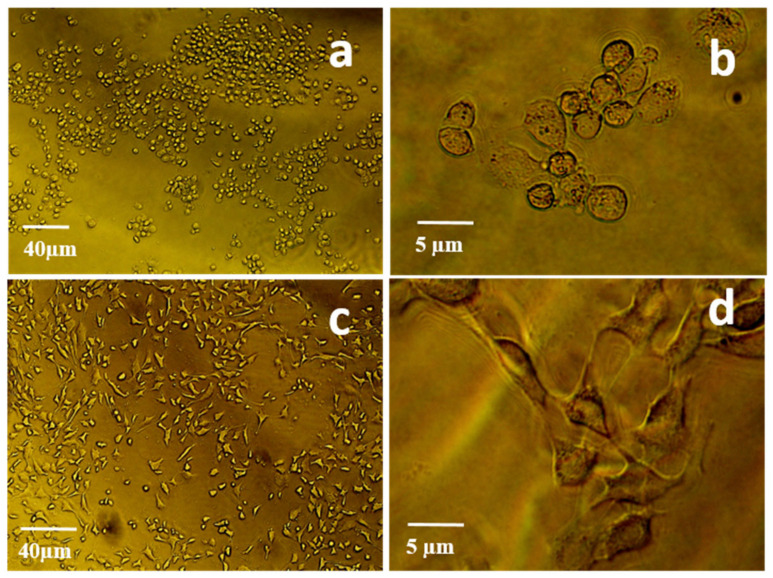

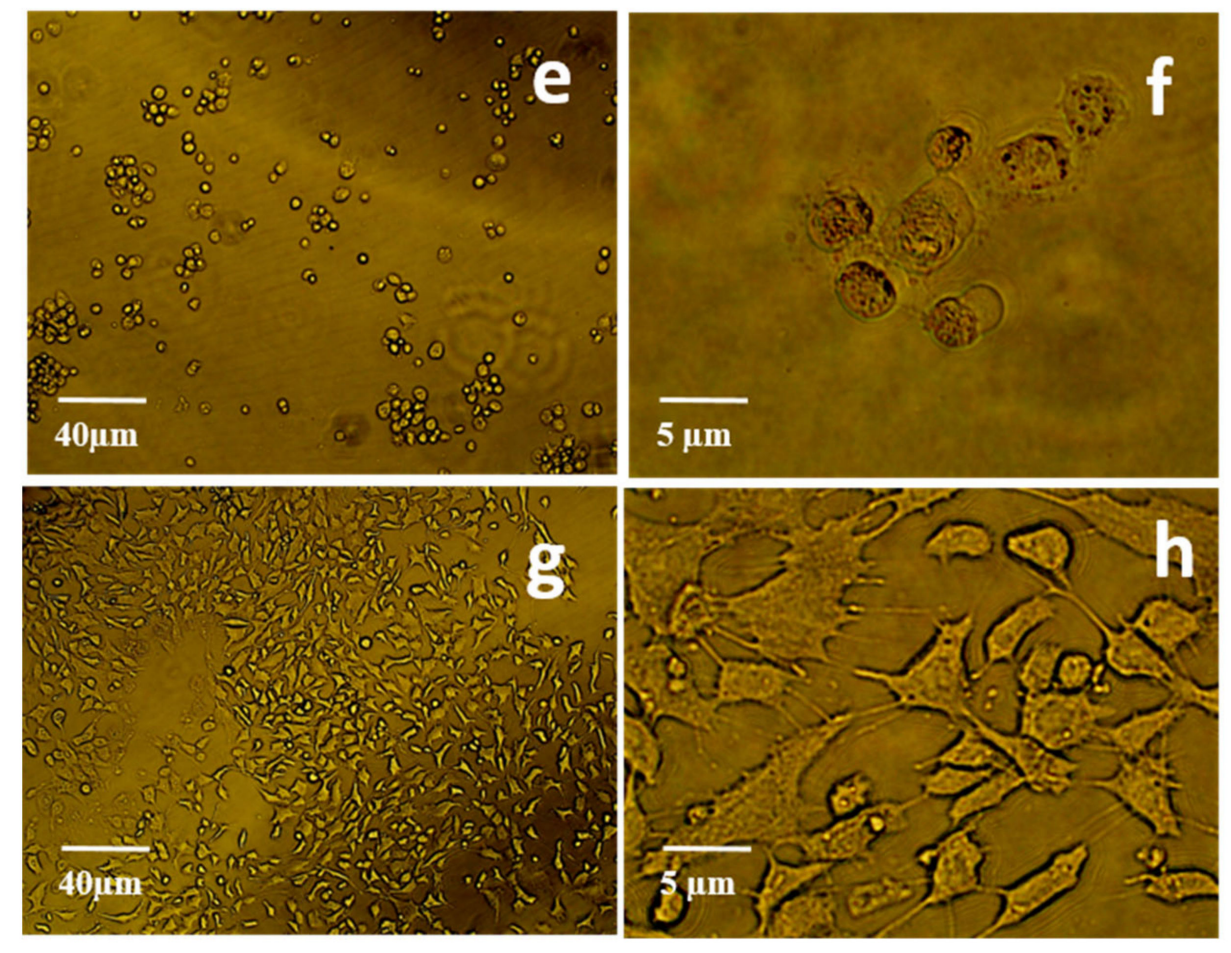
Representative microscopic images of 4T1-Luc2 cells incubated with or without aptamers. (**a**,**b**): anti-VCAM-1 ssDNA aptamers (11R6) treated cells; (**c**,**d**): anti VCAM-1 ssDNA truncated aptamers (A11R6) treated cells (**e**,**f**): anti-IL4Rα RNA aptamers (Cl42RNA) treated cells; (**g**,**h**): untreated cells.

To check whether the aptamers have the potential to promote an apoptotic effect on 4T1 tumor cells, the cells were incubated overnight with or without the aptamers in the growth media and microscopic images of the cells were captured. Cells incubated under normal conditions (Figure 3c,d) and with the truncated VCAM-1 aptamer (Figure 3g,h) appeared healthy and were found to adhere to the walls of the culture flask. However, cells incubated with either full length anti-VCAM-1 (Figure 3a,b) or anti-Il4Rα aptamers (Figure 3e,f) indicated that the aptamers might have a significant effect on the depletion of cell population in vitro. This observation was further confirmed with the absence of bioluminescence and fluorescence signals.

### 2.5. In Vivo Therapeutic Efficacy

#### 2.5.1. Noninvasive Bioluminescence Imaging

At three-weeks post-inoculation of 4T1 tumor cells and when tumor in the left mammary fat pad of Balb/c mice reached a volume of about 200 mm^3^ corresponding to a radiance efficiency of approximately 50 × 10^6^ ρ/s/cm^2^/sr, the time point was chosen to investigate the therapeutic efficacy of aptamer-conjugated SPIO nanoparticles. Three consecutives doses of SPION-11R6, SPION-A11R6 or SPION-Cl42RNA, or a combined dose of SPION-11R6 and SPION-Cl42RNA were intravenously injected. BLI and MRI in vivo monitoring were performed bi-weekly over a period of 4 weeks.

**Figure 4:**
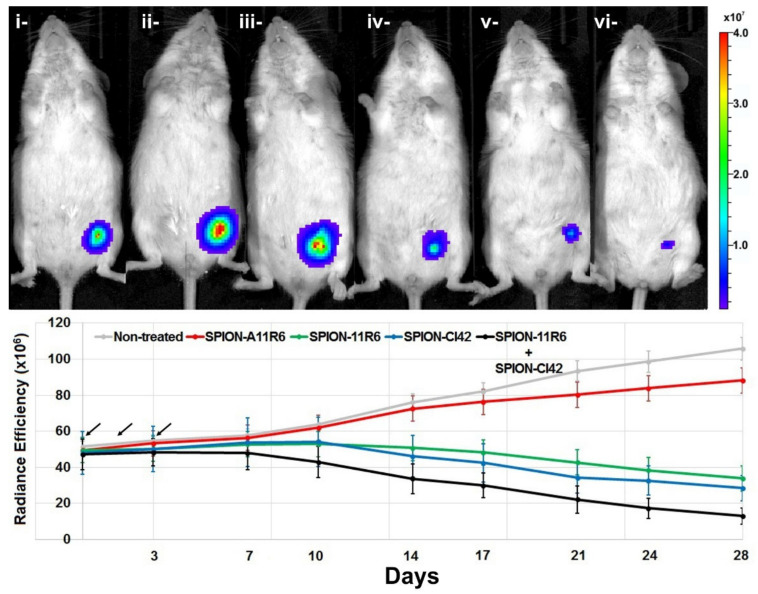
In vivo aptamer-conjugated SPIO nanoparticles therapeutic efficacy assessed using noninvasive BLI. Representative bioluminescence images (upper row) and corresponding quantitative assessments of radiance efficiency (lower row) in 4T1 tumor-bearing mice. Noninvasive imaging protocols were performed pre- (*t* = 0; corresponding to three-weeks post-tumor cell inoculation in the mammary fat pad) and up to 28 days post-injection of three consecutive doses (24 h interval time) of different aptamer therapeutic formulations. Black arrows highlight the injection time points. (**i**) pre-injection; (**ii**) non-treated mice; (**iii**) SPION-A11R6; (**iv**) SPION-11R6; (**v**) SPION-Cl42RNA; (**vi**) combined SPION-11R6 and SPION-Cl42RNA mice injected groups. Data are expressed as mean ± SD, n = 3 per group.

While the radiance efficiency in non-treated mice was found to increase gradually in a time-dependent manner to reach 105.8 ± 12.9 × 10^6^ at three-weeks investigation time point (Figure 4), the bioluminescence signal was found to be not significantly attenuated following treatment with truncated anti-VCAM-1 aptamers-conjugated SPION (SPION-A11R6). However, when mice were injected with either full length anti-VCAM-1 or anti-IL4Rα-conjugated-SPION, a significant decrease in the BLI signal was observed at the three-week investigation time point, attaining 33.9 ± 4.8 × 10^6^ and 28.4 ± 6.2 × 10^6^ (*p* < 0.05), respectively. Remarkably, this attenuation effect was significantly more prominent as, when combining both aptamer-conjugated magnetic nanoparticles, the signal decreased to 12.9 ± 2.7 × 10^6^ ρ/s/cm^2^/sr (*p* < 0.05).

#### 2.5.2. Magnetic Resonance Imaging

**Figure 5:**
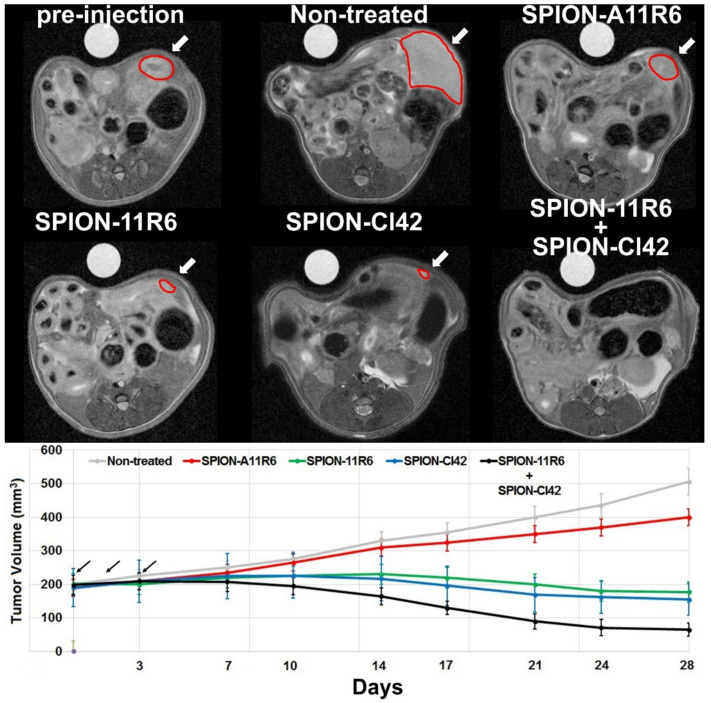
In vivo aptamer-conjugated SPIO nanoparticle therapeutic efficacy assessed using noninvasive MRI. Representative axial MR images (upper row) and corresponding quantitative measurements of tumor volume (lower row) in 4T1 tumor-bearing mice. Noninvasive imaging protocols were performed pre- (*t* = 0; corresponding to three-weeks post-tumor cell inoculation in the mammary fat pad) and up to 28 days post-injection of three consecutive doses (24 h interval time) of different aptamer therapeutic formulations. Black arrows highlight the injection time points. Pre-injection, non-treated mice, SPION-A11R6, SPION-11R6, SPION-Cl42RNA, and combined SPION-11R6 + SPION-Cl42RNA mice injected groups. White arrows reveal the tumor location and size highlighted with red contours. Data are expressed as mean ± SD, n = 3 per group.

Corroborating the BLI results, the quantification of tumor volume using MRI, as shown in Figure 5, in non-treated mice confirmed the progressive increase in tumor size with volume reaching 505 ± 65 mm^3^ (*p* < 0.05) at the end of the follow-up study. Following treatment with SPION-11R6 or SPION-Cl42RNA, a statistically significant inhibition of tumors in the mammary fat pad was observed with volume attaining 177 ± 28 (*p* < 0.05) and 155 ± 32 mm^3^ (*p* < 0.05), respectively, while the inhibition following the injection of truncated VCAM-1 aptamers (400 ± 41 mm^3^) was found to be not significant (*p* > 0.05) compared to untreated group. Similar to the observed BLI results, a synergistic and more prominent effective treatment was found when SPION-11R6 and SPION-Cl42RNA were injected (i.e., tumor volume attaining 65 ± 16 mm^3^).

## 3. Discussion

VCAM-1 and IL4Rα surface receptors specific aptamers have been reported and successfully demonstrated for the depletion of myeloid-derived suppressor cells (MDSC) and detection of inflammation in the transgenic mouse model of Alzheimer’s disease, respectively [24,25]. Selective targeting of tumor cells remains a challenging part to ensure efficient cancer therapy. Aptamers were reported as potential candidates for therapeutic applications [27]. Many aptamers are currently under investigations in clinical trials for the treatment of several diseases [28,29]. Aptamers increase biocompatibility and reduce the toxicity of magnetic nanoparticles used in biomedicine [30].

Anti-VCAM-1 and anti-IL4Rα aptamers specifically target the VCAM-1 and the IL4Rα receptors with high affinity. When the aptamers bind to the receptors, they block and inhibit their biological functions and thereby suppress the growth of the tumor cells in vitro. The results revealed that both aptamers act as anti-tumor drugs by blocking the expression of VCAM-1 and IL4Rα receptors. Gijs et al. has reported similar results for antitumor activity by monitoring the inhibitory effect of the ssDNA aptamer on over expression of HeA2 cancer cell [31]. Periostin, an extracellular matrix, is one of the overexpressed proteins in many human cancer cells which are associated with tumor growth, metastasis and angiogenesis. Benzyl-d (U)TP-modified DNA aptamer (PNDA-3) targets the FAS-1 domain of the periostin and interferes with the interaction between periostin and cell surface receptors, αvβ3 and αvβ5 integrins. PDNA-3 binding to FAS-1 deactivates the receptors and suppresses tumor cell growth and its distant metastasis [32].

A significant increase in the fluorescence intensity of the truncated VCAM-1 aptamer-treated cells was observed, indicating that the truncated aptamer neither binds to VCAM-1 receptor nor consequently affects the metabolic activity of the cells. This could be attributed to the lack or poor interaction of the truncated aptamer into the binding pocket of the VCAM-1 receptor. The IL4Rα specific aptamer showed a higher effect on the depletion of cell growth compared to the VCAM-1 aptamer. The difference in the number of dead cells in the presence of the two different aptamers was presumably due to the interaction of the aptamers to their specific target repair mechanisms. Interestingly, the reported affinity constants of the IL4Rα specific aptamer and VCAM-1 aptamer are 14 nM and 49 nM in vitro, respectively [24,25]. IL4Rα specific aptamer has high affinity towards to its target. Though consistent with the depletion of cell deaths, this might be one of the possible factors, in addition to other mechanisms of aptamer action on the targets, for the depletion in the cell counts.

Upon the binding of aptamers to their respective VCAM-1 and IL4Rα receptors, they block their biological function and thus promote cancer cell deaths. This was clearly indicated by the complete absence of luminescence upon incubating 4T1-Luc2 cells with 11R6 and C142RNA aptamers. VCAM-1 overexpressed on the surface of cancer cells binds with the counter receptor α4β1integrin and activated phosphoinositide 3-kinase activity, which led to the further survival of the cancer cells. Anti-α4 integrin inhibitors have been developed for the prevention of VCAM-1 and α4β1 interactions. On the other hand, the inhibition of the VCAM-1 function with anti-VCAM aptamers may have similar effects. In vitro selection of VCAM-1 ssDNA aptamers has been reported and applied for early stage detection of cerebrovascular inflammation [23].

Though the IL4Rα is expressed in MDSC and TAM, it can also be found in most hematopoietic and non-hematopoietic cells [25]. IL4Rα expressed on the 4T1-Luc2 cells surfaces were well-recognized by the anti-IL4Rα aptamer with similar affinity. The aptamer specifically binds to IL4Rα receptor overexpressed on the tumor-bearing mice cells and it was found not only to inhibit the primary tumor cells, but also to deplete neoplastic cells in the lung. These effects are due to the specific binding of the aptamer to IL4Rα, which was confirmed by the treatment with non-specific DNA oligonucleotides.

The in vivo therapeutic efficacy evaluated in tumor-bearing mice confirmed the therapeutic efficacy of anti-VCAM-1 and anti-IL4Rα aptamers that were conjugated to SPION as assessed by monitoring tumor growth and inhibition using noninvasive BLI and MRI. A synergistic and more prominent therapeutic effect was observed when combining both anti-VCAM-1 ssDNA and anti-IL4Rα RNA aptamers.

## 4. Materials and Methods

*N*-hydroxysuccinimide (NHS) and 1-(3-dimethylaminopropyl)-3-ethyl-carbodiimide (EDC) were purchased from Sigma-Aldrich (Saint-Louise, MO, USA). Analytical grade 2-amino-2-(hydroxymethyl)-1,3-propanediol (Tris base), bovine serum albumin (BSA), ethylenediaminetetraacetic acid (EDTA), sodium azide, potassium phosphate and sodium chloride from Sigma-Aldrich (Saint-Louise, MO, USA) were used in these experiments. HPLC purified labeled and functional oligonucleotides (Table 1) were obtained from Metabion International (Planegg, Germany). Carboxylic acid-functionalized polyethylene glycol (PEG 2000) coated SPIO nanoparticles were supplied by Micromod Partikeltechnologie GmbH (Rostock, Germany). They have a hydrodynamic size of 132.5 ± 5.9 nm and a surface charge of 2.71 ± 0.62 mV, based on measurement of the zeta potential using a Zetasizer Nano ZS90 (Malvern Instruments, Malvern, UK). D-Luciferin Firefly and potassium salt was purchased form PerkinElmer (Waltham, MA, USA). Aptamer conjugated SPIO magnetic beads were characterized by FT–IR spectroscopy using Thermo Fisher Scientific Nicolet Si10 (Waltham, MA, USA). The ssDNA and RNA aptamers were dissolved in ultrapure Milli-Q water to make the stock solutions and stored at −20 °C until further use. The DNA solutions used in the experiments were diluted in the corresponding buffer. Roswell Park Memorial Institute (RPMI 1640) medium, heat-treated fetal bovine serum (FBS) 0.25% trypsin and penicillin–streptomycin were purchased from Gibco, Life Technologies (Carlsbad, CA, USA). Scepter 2.0 Cell Counter from Millipore (Billerica, MA, USA) was used for cell counting.

## 4.1. Cancer Cell Culture

Luciferase expressing 4T1 murine breast cancer cells (4T1-Luc2) were purchased form PerkinElmer and cultured as reported previously [33]. The bioluminescent 4T1-Luc2 cells act as a sensor for their in vivo and in vitro expression to monitor tumor proliferation in animals. Cells were cultured in RPMI 1640 medium containing 10% heat-treated fetal bovine serum (FBS) and 100 unit/mL penicillin-streptomycin (Gibco, Life Technologies, Carlsbad, CA, USA) in the incubator at 37 °C in a humidified atmosphere containing 5% CO_2_.

## 4.2. Cell Counting

After 75% confluence, the cells adhering on the walls of the culture flask were detached by incubating cells with 2 mL of 0.25% trypsin at 37 °C. After 7 min, 8 mL of media containing FBS was added and centrifuged at 1100 rpm for 10 min. The supernatant was discarded, and the pellet of the cells was dispersed in PBS buffer. The number of cells in the solutions was measured using automated cell counter.

## 4.3. Preparation of Aptamer-Conjugated SPIO Nanoparticles

SPIONs were prepared by the core-shell method containing 75–80% (*w/w*) magnetite with shell dextran followed by incubation with the mixture of PEG, diglycidyl ether and epichlorohydrin in the pH range of 11 to 12 for 24 h at room temperature [34]. Ready to use SPIONs were stored in deionized water after magnetic separation. The carboxylic acid functionalized magnetic nanoparticles were characterized, and their biocompatibility have been previously reported [34,35]. The PEG (2000) coated SPIO nanobeads were washed with water and sonicated for 5 min with a frequency of 35 Hz at 37 °C, followed by washing with coupling buffer until the supernatant was clear. A mixture of 2 µM 5′-amine functionalized anti-VCAM-1 ssDNA aptamers (11R6, A11R6) or anti-IL4Rα (Cl42RNA) RNA aptamers, SPIO magnetic beads suspension and coupling agent (100 mM EDC and 25 mM NHS) in coupling buffer (10 mM potassium phosphate, 150 mM sodium chloride, pH 4.5–5.0) were mixed for 4 h at room temperature. The unreacted aptamers were removed by washing the beads with wash buffer. The active groups on SPIO nanomagnetic beads were blocked by incubating with 10 mM Tris, 150 mM sodium chloride, 0.1% (*w/v*) BSA (pH 7.5) for one hour and stored in 10 mM tris buffer with 0.05% sodium azide (pH 7.5) at 4 °C until further use. The concentration of the aptamer-conjugated beads was adjusted to 2.5 mg/mL.

## 4.4. Cell Viability Assay

NanoDrop 2000C spectrophotometer was used to measure the absorption of Alamar blue dye and DNA/RNA quantification. The fluorescences of samples were measured using Nanodrop ND3300 fluorospectrometer (Thermo Fisher Scientific) and/or Molecular device F5 fluoromax microtiter plate reader (Sunnyvale, CA, USA) using 96-well plates. UV–Visible absorption and fluorescence intensity changes were used to calculate the oxidized or reduced form of Alamar blue.

The viability of 4T1-Luc2 cells in the presence of 5 µM anti-VCAM-1 ssDNA or anti-IL4Rα RNA aptamers was assessed by monitoring the changes in the absorbance and the fluorescence of alamar blue, according to the manufacturer’s instructions (Thermo Fisher Scientific). Cells at equal amounts (5000 cells/mL) were seeded in 96-well plates and incubated overnight at 37 °C in a humidified atmosphere containing 5% CO_2_. Then, 10% of Alamar blue from the stock solution was added to each well. Change in the absorbance at 570 nm was monitored at 0, 3.5, 5.5 and 24 h. The fluorescence intensities (Ex/Em = 595/635 nm) were measured and the percentage increase in the fluorescence intensity with time was plotted. The percentage reductions in the Alamar blue from the absorption and the fluorescence were calculated from equation:

%Reduction = [(εOX)λ2 × Aλ1 − (εOX)λ1 × Aλ2/(εRED)λ1 × A′λ2 − (εRED)λ2 × A′λ1] × 100

where:

ε_OX_ = molar extinction coefficient of the oxidized form of Alamar blue
ε_RED_ = molar extinction coefficient of the reduced form of Alamar blue
A = absorbance of test wells
A′ = absorbance of negative control well (the negative control well contain media + Alamar blue without cells)
λ_1_ = 570 nm; λ_2_ = 600 nm

## 4.5. In Vitro Bioluminescence Assay

Equal amounts of 4T1-Luc2 cells (5000 cells/mL) were seeded in a 96-well plate overnight with 5 µM solution of anti-VCAM-1 ssDNA or anti-IL4Rα RNA aptamers. Before the measurement, the medium was removed and washed with pre-incubated (37 °C) PBS. Pre-warmed media with 150 µg/mL of D-luciferin was added to each well and the bioluminescence was monitored at 37 °C in real time every 30 s. The luminescence intensity was plotted and the viability of the cells was calculated with respect to the control.

## 4.6. Animal Preparation and In Vivo Study Design

All animal procedures were performed in accordance with the National guidelines for the care of laboratory animals and the study was approved by the Ethical Committee of the College of Applied Medical Sciences (agreement number: CAMS06/3334). Female Balb/c mice (16–18 g) were retained in a temperature-regulated ventilated cabinet and provided free access to sterilized food and water on a 12-h light/dark cycle. During the various protocols, each animal was anesthetized by an intraperitoneal (i.p.) administration of a mixture of 0.1 mL of 4 mL of ketamine (50 mg/mL), 1 mL of xylazine (2%), and 5 mL of physiological saline.

The tumor model was established by injecting 10^6^ 4T1-Luc2 cancerous cells in the left inguinal mammary fat pad of Balb/c mice. At three-weeks post-inoculation, the tumor-bearing mice (*n* = 3 per condition) were injected with three consecutive doses (24h interval time) of either 100 μL of physiological saline (non-treated group), 100 μL of either SPION-11R6, SPION-A11R6 or SPION-Cl42RNA, or a combined dose of 50 μL of SPION-11R6 and 50 μL of SPION-Cl42RNA.

## 4.7. Noninvasive Bioluminescence Imaging

Tumor-bearing mice underwent biweekly bioluminescence imaging using an IVIS Lumina Series III preclinical in vivo imaging system (PerkinElmer) to monitor the growth of the tumor site. The therapeutic efficacy was then longitudinally monitored following each treatment condition for up to 4 weeks. D-Luciferin firefly substrates (150 mg Luciferin/kg body weight) were intraperitoneally injected 10 min before imaging according to the manufacturer’s protocol. The radiance efficiency was assessed by measuring the photon flux at the different investigation time-points during the follow-up study.

## 4.8. Magnetic Resonance Imaging

Tumor volume in the mammary fat pad before and after treatment was also quantified using noninvasive MRI on a 4.7T Pharmascan 47/16 Bruker magnet interfaced to ParaVision5.1 (Bruker Biospin GmbH, Rheinstetten, Germany). Fast spin echo RARE (rapid acquisition with refocused echoes) sequence with TR/TE = 2500/10 ms, RARE factor = 8, 4 averages and 100 × 100 μm in plane resolution was applied, as previously described [36]. Continuous 1 mm thick slices were used to cover the entire tumor region.

## 4.9. Statistical Analyses

Data are presented as mean ± SD. Statistical significance was evaluated using ANOVA followed by Bonferroni–Dunn post-hoc test using SPSS v12.0 (SPSS Inc., Chicago, IL, USA) software. Two-way ANOVA was done to determine if significant differences existed in the means between different groups. Values of *p* < 0.05 were considered statistically significant.

## 5. Conclusions

In summary, anti-VCAM1 and anti-IL4Rα aptamers specific to VCAM-1 and IL4Rα receptors biomarkers that are overexpressed in 4T1-Luc2 tumor bearing mice were used as diagnostic and therapeutic tools for breast cancer. The cell viability and the bioluminescence assay confirmed that these specific aptamers suppressed the function of VCAM-1 and IL4Rα receptors and promoted the apoptosis of 4T1-Luc2 cells. The anti-IL4Rα aptamers were found to inhibit cell growth more efficiently compared to anti-VCAM-1 aptamers. The therapeutic efficacy of the combined anti-VCAM-1 ssDNA and anti-IL4Rα RNA aptamers in tumor-bearing mice was more pronounced compared with either anti-VCAM-1 ssDNA or anti-IL4Rα RNA aptamer, as assessed by monitoring tumor growth and inhibition using noninvasive BLI and MRI. Based on the above observations, the combination treatment of these aptamers, following conjugation with SPION offers a potential approach for enhanced diagnosis and therapy of breast cancer.

## Figures and Tables

**Table 1:** ssDNA and RNA Aptamers Used in This Study.

Name	Sequence (5′to 3″)
11R6	ATACCAGCTTATTCAATTGGACACGGCAAAGGGGTATAGCCTACCGGACCGTGAACATGGAATGGTGTGCTGCGTGGAGATAGTAAGTGCAATCT-3
Am-11R6	H_2_NATACCAGCTTATTCAATTGGACACGGCAAAGGGGTATAGCCTACCGGACCGTGAACATGGAATGGTGTGCTGCGTGGAGATAGTAAGTGCAATCT-3
A11R6	GGACACGGCAAAGGGGTATAGCCTACCGGACCGTGAACATGGAATGGTGTGCTGCGTGG
Am-A11R6	H_2_N-GGACACGGCAAAGGGGTATAGCCTACCGGACCGTGAACATGGAATGGTGTGCTGCGTGG
Cl42RNA	AAAAAGCAACAGGGUGCUCCAUGCGCAUGGAACCUGCGCG
Am-Cl42RNA	H_2_N- AAAAAGCAACAGGGUGCUCCAUGCGCAUGGAACCUGCGCG

## Supplementary Materials

The following are available online, Figure S1: IR spectrum of the SPIONs.
