# Supplementary material for: Anti-VCAM-1 and Anti-IL4Rα Aptamer-Conjugated Super Paramagnetic Iron Oxide Nanoparticles for Enhanced Breast Cancer Diagnosis and Therapy
Source: Molecules. 2020 Jul 29;25(15):3437. doi: 10.3390/molecules25153437 (PMC7435411; doi:10.3390/molecules25153437)
Supplement: Supplementary file 1 [file molecules-25-03437-s001.pdf]

Anti-VCAM-1 and anti-IL4Rα aptamer conjugated super paramagnetic iron oxide nanoparticles for enhanced breast cancer diagnosis and therapy

Raja Chinnappan[1], Achraf Al Faraj[2*], Anas Abdulrahman[3], Khalid M. Abu-Salah[4], Fouzi Mouffouk[5], Mohammed Zourob[1, 3, *]

[1] Department of Chemistry, Alfaisal University, Al Zahrawi Street, Al Maather, Al Takhassusi Rd, Riyadh 11533, Saudi Arabia

[2] Department of Radiologic Sciences, Faculty of Health Sciences, American University of Science and Technology, Ashrafieh, Alfred Naccash Avenue, Beirut 1100, Lebanon

[3] King Faisal Specialist Hospital and Research Center, Zahrawi Street, Al Maather, Riyadh 12713, Saudi Arabia

[4] Department of Nanomedicine, King Abdullah International Medical Research Center/ King Saud bin Abdulaziz University for Health Sciences, King Abdulaziz Medical City, Riyadh 11481, Saudi Arabia

[5] Department of Chemistry, Kuwait University, P.O. Box 5969, Safat, 13060 Kuwait.

Characterization of SPIONs:

Aptamers with amine functional group at the 5′ ends were coupled with carboxylic acid functionalized PEGylated SPION magnetic nanoparticles by EDC/NHC chemistry. 100mM EDC and 25 mM NHS were used as coupling agent in aqueous medium to link the SPIONs and aptamers. The conjugation of the aptamers on the surface of the PEGylated SPIONs were characterized by IR spectroscopy. The IR vibration frequency of carbonyl group from 1725 cm^{-1} to 1650 cm^{-1} As shown in figure S1. After conjugation, the carboxylic acids were converted to amide and the IR absorption at 1725 cm^{-1} and 1650 cm^{-1} were corresponding to the carbonyl group in the carboxylic acid and amide, respectively [26]. The increase in the intensity at 1650 is the convertion of carboxylic acid to the amide after aptamer conjugation.

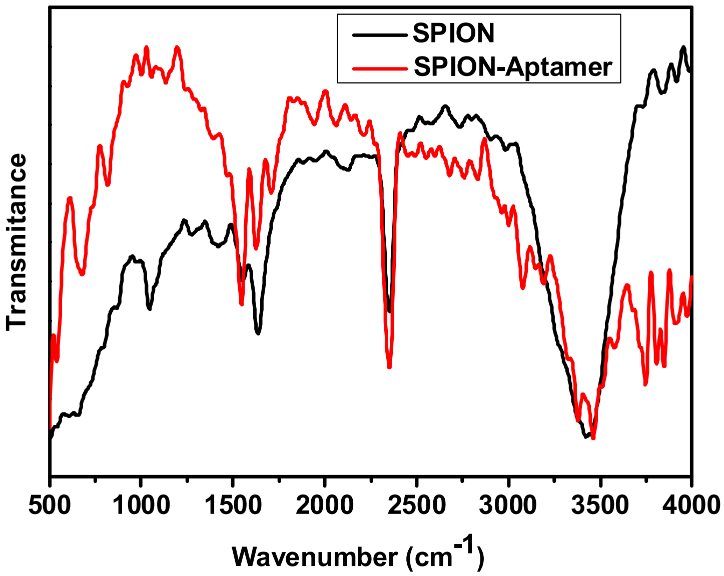

Figure S1: IR spectrum of the SPIONs: The change in the ratio between the intensities of the vibrational frequencies at 1725 cm^{-1} and 1650 cm^{-1} of the SPIONs was observed (i.e. conversion of carboxylic acid into amide).
